# Experimental Study on the Compatibility of PD Flexible UHF Antenna Sensor Substrate with SF6/N2

**DOI:** 10.3390/mi14081516

**Published:** 2023-07-28

**Authors:** Xukun Hu, Guozhi Zhang, Guangyu Deng, Xuyu Li

**Affiliations:** 1 Hubei Engineering Research Center for Safety Monitoring of New Energy and Power Grid Equipment, Hubei University of Technology, Wuhan 430068, China; 2010221106@hbut.edu.cn (X.H.); 102110311@hbut.edu.cn (G.D.); 2010231403@hbut.edu.cn (X.L.); 2 School of Electrical and Electronic Engineering, Hubei University of Technology, Wuhan 430068, China; 3 State Grid Electric Power Research Institute Wuhan Nanrui Co., Ltd., Wuhan 430074, China

**Keywords:** flexible UHF antenna sensor, flexible base, DC gas-insulated switchgear (GIS), compatibility, SF6/N2 gas mixture

## Abstract

The use of flexible, built-in, ultra-high-frequency (UHF) antenna sensors is an effective method to solve the weak high-frequency electromagnetic wave signal sensing of partial discharge (PD) inside gas-insulated switchgears (GISs), and the compatibility of flexible UHF antenna sensor substrate materials and SF6/N2 mixtures is the key to the realization of a flexible UHF antenna sensor inside a GIS. Based on this, this paper builds an experimental platform for the compatibility of a 30% SF6/70% N2 gas mixture and a PD flexible UHF antenna sensor substrate and conducts compatibility experiments between the 30% SF6/70% N2 gas mixture and PD flexible UHF antenna sensor substrate under different temperatures in combination with the actual operating temperature range of the GIS. In this article, a Fourier transform infrared spectrometer, scanning electron microscope and X-ray photoelectron spectrometer were used to test and analyze the gas composition, the surface morphology and the elemental change in the PD flexible UHF antenna sensor substrate, respectively. PET material will be slightly oxidized under the environment of a 30% SF6/70% N2 gas mixture at 110 °C, PI material will generate metal fluoride under the environment of a 30% SF6/70% N2 gas mixture and only PDMS material will remain stable under the environment of a 30% SF6/70% N2 gas mixture; therefore, it is appropriate to use PDMS substrate in the development of flexible UHF antenna sensors.

## 1. Introduction

SF_6_ is widely used in insulation systems of electric equipment such as gas-insulated switchgears (GISs) because of its good insulating ability and arc-extinguishing ability. However, SF_6_ has a large greenhouse effect, its decomposition products are toxic and corrosive and its cost is high. Therefore, in order to reduce the use of SF_6_ gas, the most effective method is to develop the use of SF_6_ gas mixtures. Current research shows that SF_6_ mixed with N_2_ has a good synergistic effect and its insulation performance is stronger than N_2_ and more similar to SF_6_ [1]. In recent years, the State Grid Corporation, in response to the national call for energy savings and emission reductions, organized work on the SF_6_/N_2_ gas mixture for GIS busbars and isolation and grounding switches, and they also determined the use of a 30% SF_6_/70% N_2_ gas mixture as an insulation medium for GIS busbars and isolation and grounding switches [2]. Partial discharge (PD) is the main cause of GIS insulation failure, and if not dealt with in a timely manner, it will likely lead to serious equipment accidents and grid accidents [3,4,5]. The ultra-high-frequency (UHF) method uses antenna sensors to sense the high-frequency electromagnetic wave signals radiated by PD, which has the advantages of strong anti-interference capability and high reliability, and it is widely used for the detection of GIS PD insulation defects [6,7]. At present, all newly commissioned GISs of 220 kV and above in China’s power grid companies are installed with UHF detection systems or reserved with UHF detection interfaces. According to the installation position, UHF antenna sensors for GIS PD detection can be divided into two types: built-in and external [8,9,10]. External UHF antenna sensors will be affected by the serious attenuation of electromagnetic wave signals caused by outward PD leakage due to the GISs’ own metal shell structure and the influence of the corona interference signal and mobile communication signal in the external environment, resulting in inadequacies in its sensitivity to the PD in the GIS, especially in intermittent PD signal sensing. Although built-in UHF antenna sensors can effectively overcome the shortcomings of external UHF antenna sensors, most of the current antenna sensors for UHF detection built into GISs are made of rigid materials such as those with FR-4 epoxy resin as the substrate [11,12,13], which cannot conform to the cylindrical metal structure housing of GISs and require the complex structural modification of the flange of the device itself. In addition, rigid substrates in built-in UHF antenna sensors also create the risk of problems such as the electric field distribution inside the device being affected [14].

For the creation of flexible, built-in GIS PD detection UHF antenna sensors, our group has developed a variety of UHF GIS PD detection antennas based on two PD flexible UHF antenna sensor substrates, namely thermosetting polyimide (PI) and polydimethylsiloxane (SU-8/PDMS). These flexible antennas have obvious advantages such as ultra-small sizes (maximum sizes of less than 60 mm), ultra-thinness (thicknesses of up to 0.2 mm), excellent radiation performances and stable performances in the bending radius deformation range of 150–500 mm [15,16,17,18].

Because the process of building a flexible UHF antenna sensor into a GIS puts it in direct contact with a SF_6_/N_2_ gas mixture, if the flexible UHF antenna sensor is incompatible with SF_6_/N_2_ inside the equipment, on the one hand, this may cause the PD flexible UHF antenna sensor substrate material to be corroded by the gas mixture, which will lead to problems such as the inaccurate detection of PD signals by the flexible antenna sensor; on the other hand, the flexible UHF antenna sensor may lead to the decomposition of the mixed gas, resulting in the degradation of the insulation performance of the mixed gas, and then causing more serious insulation accidents. At the same time, it is still unclear which built-in UHF antenna sensor substrate material has better compatibility with the SF_6_/N_2_ gas mixture, which is directly related to the safety of the long-term operation of built-in flexible UHF antenna sensors in GISs and the field implementability of the technology.

Based on this, an experimental study on the compatibility of PD flexible UHF antenna sensor substrates with SF_6_/N_2_ is proposed in this paper. Firstly, the experimental platform used to determine the compatibility of the SF_6_/N_2_ gas mixture and the flexible UHF antenna sensor substrate material was built, and thermal acceleration experiments were carried out with the SF_6_/N_2_ gas mixture and three commonly used PD flexible UHF antenna sensor substrate materials at different temperatures, according to the actual operating temperature of a GIS. The results are summarized and analyzed to enable us to select the most suitable PD flexible UHF antenna sensor substrate materials and provide basic reference data for the design and engineering application of GIS PD flexible built-in UHF antenna sensors.

## 2. Compatibility Experimental Platform and Experimental Materials

### 2.1. Compatibility Experimental Platform

**Figure 1:**
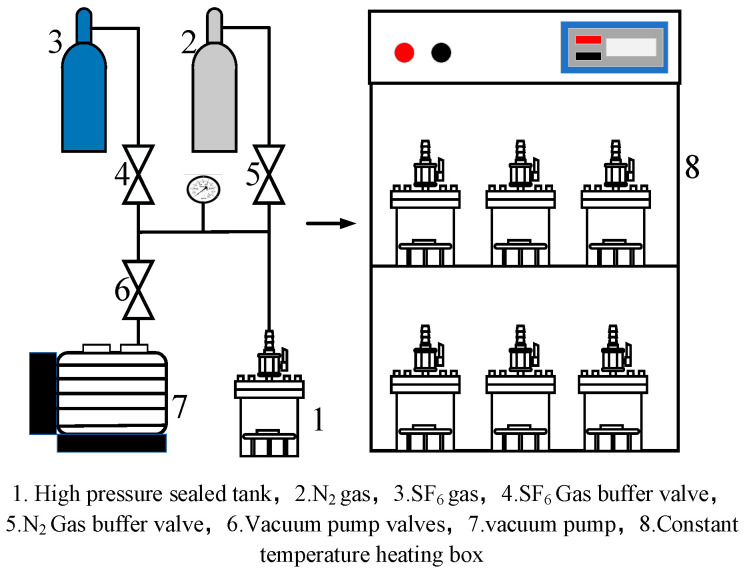
Experimental platform.

The thermal acceleration experimental platform is shown in Figure 1. The experimental platform was mainly composed of a temperature control system and a switching system. The experimental temperature was regulated by the temperature control system at the beginning of the experiment, and the temperature inside the chamber was set using the internal detection system. The high-pressure sealed tank shell was made of stainless steel, and a stainless steel mesh holder was placed in the gas chamber for the PD flexible UHF antenna sensor substrate material, which was intended to be in full contact with the SF_6_/N_2_ gas mixture and to ensure uniform heating of the sample. The high-pressure sealed tank was cleaned with SF_6_ gas several times before the experiment; the pressure value in the gas chamber was checked using a barometer during inflation; and the exhaust-gas-collection device was used to collect the excess gas and reduce the pollution to the environment.

### 2.2. Experimental Materials

**Figure 2:**
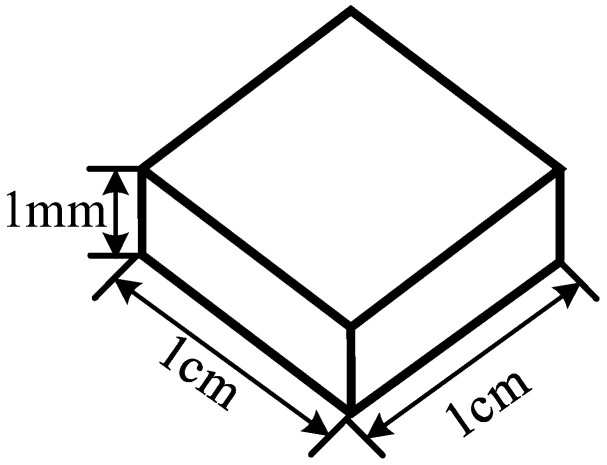
Sample of flexible material.

Due to the continuous development of flexible wearable devices, flexible antennas have become a research hotspot in recent years. Antennas with flexible materials as substrates have good deformation capabilities and can be co-profiled with complex structures, so the selection of suitable flexible substrates is the key to the realization of the development of flexible UHF antenna sensors. Common flexible dielectric substrates mainly include PI, polyethylene terephthalate (PET) and PDMS as PD flexible UHF antenna sensor substrate materials. These materials are bendable, have high tensile strength, are non-toxic, have high temperature resistance, have good insulation properties, have low dielectric constants and have good dimensional stability. It is these excellent properties that make these materials ideal for making flexible UHF antenna sensor substrates [19,20]. Therefore, in this paper, three PD flexible UHF antenna sensor substrate materials, PI, PET, and PDMS, were selected for the study to investigate their compatibility with SF_6_/N_2_. The dimensions of the experimental samples of the flexible materials used for accelerated thermal aging are shown in Figure 2.

## 3. Experimental Method

According to GB/T11022-2020 [21], the maximum allowable temperature of the accessible parts of the equipment is 70 °C, and DL/T 617-2010 states that the maximum temperature rise inside the cavity is 40 K. Therefore, in order to investigate the compatibility of the SF_6_/N_2_ gas mixture with the PD flexible UHF antenna sensor substrate material under the actual operating temperature and the temperature limit of the equipment, the experimental temperatures were set to 40 °C, 70 °C and 110 °C, respectively. The temperature was controlled by using a constant temperature heating chamber. The experimental sample specimens were placed in the SF_6_/N_2_ gas mixture environment, and the thermal acceleration experiments were continuously carried out under three different temperatures for 7 days and 24 h per day.

According to GB/T11021-2014, “Electrical Insulation, Heat Resistance and Representation Method” and “Evaluation and Identification of Electrical Insulation System: ‘IEC60505:2011’”, according to the 6-degree rule, the compatibility of the set experimental temperature range and experimental time can better simulate the service life of gas-insulated equipment under normal working conditions.

(1) $$\mathrm{Equivalent}\;\mathrm{formula}:\;t_{2}=2^{\frac{T_{1}-T_{2}}{6}}\times t_{1}$$

Among them, *t*_1_ represents the duration of the thermal acceleration experiment, t_2_ represents the equivalent duration of the GIS operating temperature, *T*_1_ represents the experimental temperature and *T*_2_ represents the GIS operating temperature. It is calculated that when the experimental duration is 7 days and the experimental temperature is 110 °C, the maximum ambient temperature of GIS operation is 40 °C, and the equivalent duration is calculated to be 62.34 years [22].

### Experimental Steps

**Figure 3:**
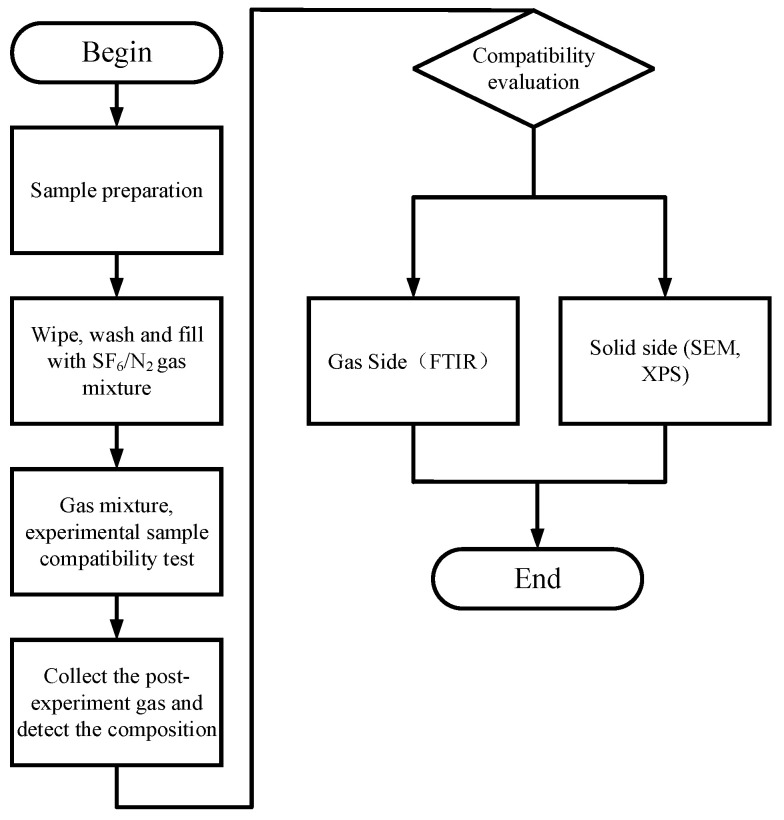
Flowchart of experimental steps.

The experimental steps are shown in Figure 3. Before the experiment, the surface of the small experimental sample and the inner wall of the experimental gas tank were wiped with absolute ethanol, and after the sample and the tank were naturally air-dried, the experimental sample was put into the bottom bracket of the gas tank. The experimental gas tank had good air tightness, could withstand a maximum pressure of 0.8 MPa, and had a capacity of about 400 mL. Before the experiment, SF_6_ was used to wash and vacuum the gas chamber, and the above operation was repeated 3 times to avoid impure gas affecting the results. The Dalton partial pressure law was used to fill the gas tank with 0.5 MPa 30% SF_6_/70% N_2_ gas, put the gas tank into a constant temperature heating box for the heating treatment for 7 days and set up room-temperature (20 °C) experimental control groups. After the experiment, the composition of the mixed gas was analyzed using a Fourier transform infrared spectrometer (FTIR), and a scanning electron microscope (SEM) and X-ray photoelectron spectroscopy (XPS) were used to test and analyze the surface morphology and elemental changes in the PD flexible UHF antenna sensor.

## 4. Experimental Results and Analysis

### 4.1. Analysis of the Influence of PD Flexible UHF Antenna Sensor Substrate on SF_6_ Gas Itself

An FTIR is an infrared spectrometer developed based on the principle of the Fourier transform of infrared light after interference, which is mainly composed of an infrared light source, diaphragm, interferometer (beam splitter, moving mirror and fixed mirror), sample chamber, detector and various infrared mirrors, lasers, control circuit boards and power supplies. Fourier infrared spectroscopy can process a signal via Fourier transform to obtain an infrared absorption spectrum containing absorbance with a wavenumber. At the same time, since the chemical vibrations of different functional groups have different absorption characteristics of infrared light with different wavenumbers, the qualitative and quantitative analysis of SF_6_/N_2_ properties can be realized by comparing the FTIR infrared absorption spectra before and after the experiment.

**Figure 4:**
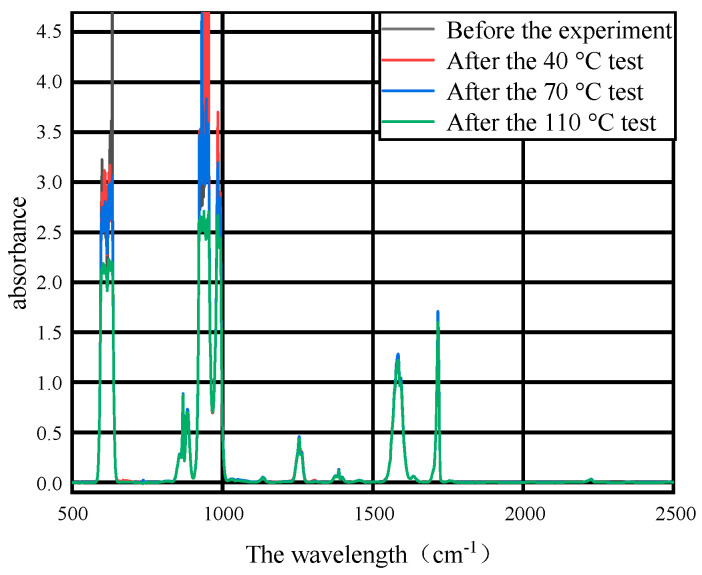
Infrared spectra of gas after PI and SF_6_/N_2_ tests at different temperatures.

**Figure 5:**
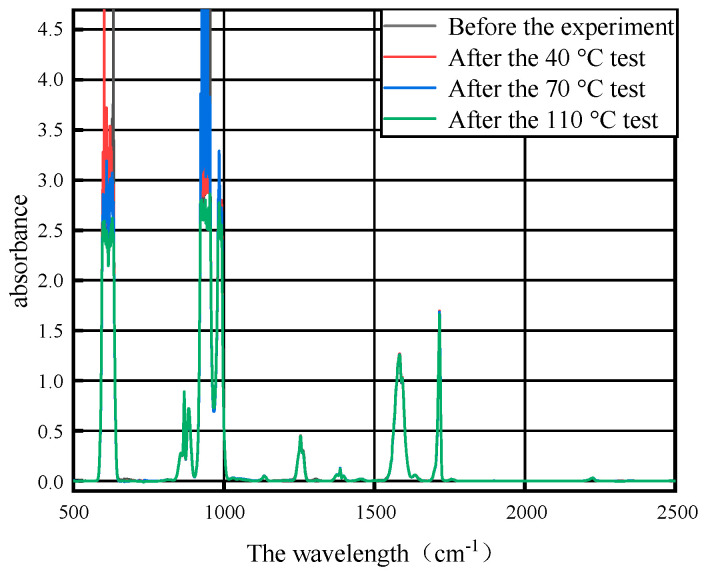
Infrared spectra of gas after PET and SF_6_/N_2_ tests at different temperatures.

**Figure 6:**
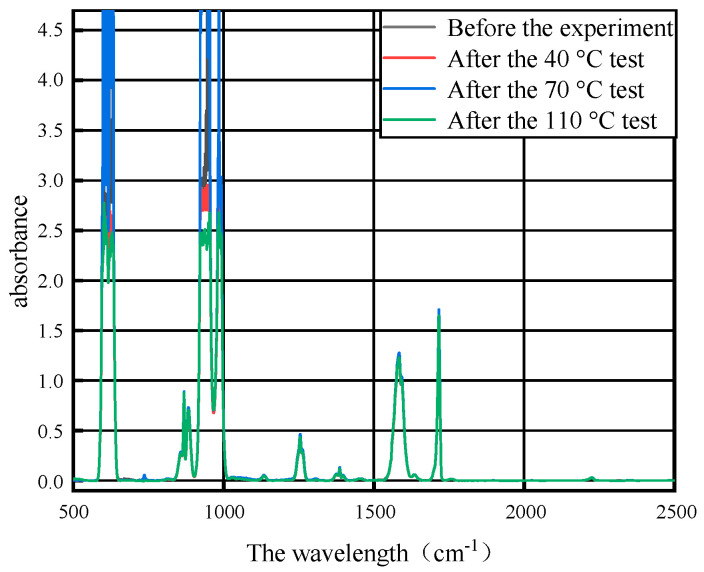
Infrared spectra of gas after PDMS and SF_6_/N_2_ tests at different temperatures.

After the 7-day thermal acceleration experiment of 40 °C, 70 °C and 110 °C, respectively, on the base materials of the three PD flexible UHF antenna sensor substrates of PI, PET and PDMS, the mixed gas after the gas collection experiment was used for FTIR detection, and the FTIR detection results of the mixed gas before and after the experiment of the base material of PI, PET and PDMS for three PD flexible UHF antenna sensors are shown in Figure 4, Figure 5 and Figure 6.

It can be seen from Figure 4, Figure 5 and Figure 6 that the SF_6_/N_2_ mixture in the control group peaks in the infrared spectrum with the SF_6_/N_2_ mixture after the experiment, which indicates that the composition of the mixed gas does not change after the experiment, and the base materials of the three PD flexible UHF antenna sensors of PI, PET and PDMS will not affect the SF_6_/N_2_ mixture within the GIS operating temperature range.

### 4.2. Analysis of the Influence of SF_6_/N_2_ Mixture on PD Flexible UHF Antenna Sensor Substrate

If the SF_6_/N_2_ mixture reacts with the PD flexible UHF antenna sensor substrate material, it may not only cause the SF_6_/N_2_ mixture to decompose but also cause corrosion to the PD flexible UHF antenna sensor substrate material and introduce new uncertain risk factors.

#### 4.2.1. PD Flexible UHF Antenna Sensor Substrate Topography Detection

**Figure 7:**
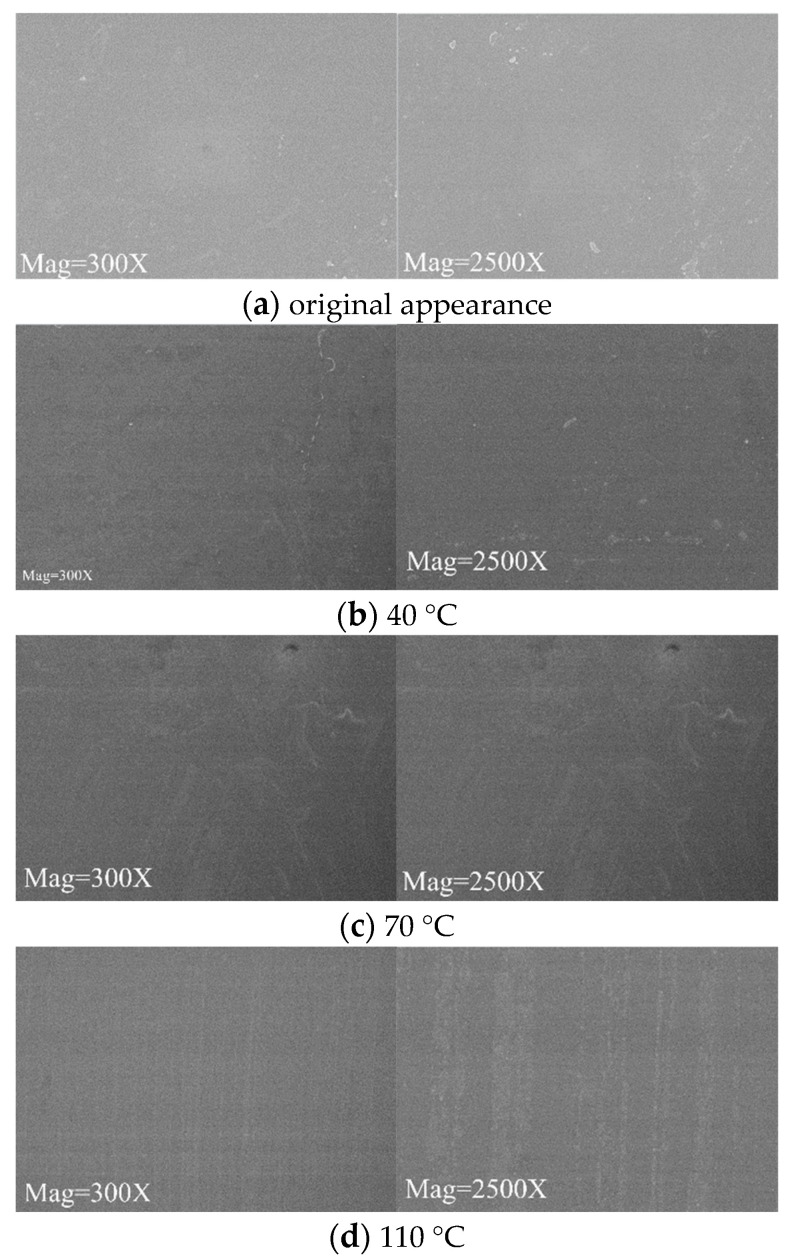
SEM of PI material.

**Figure 8:**
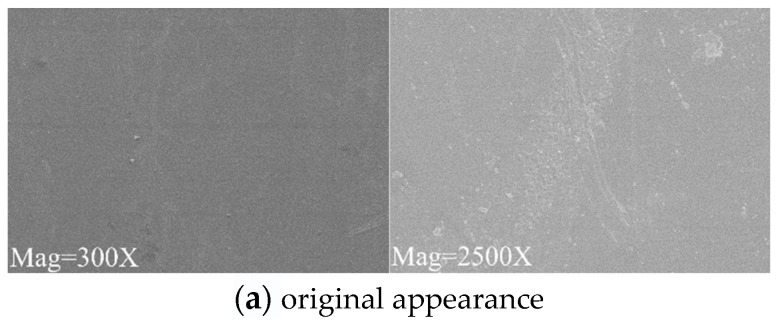

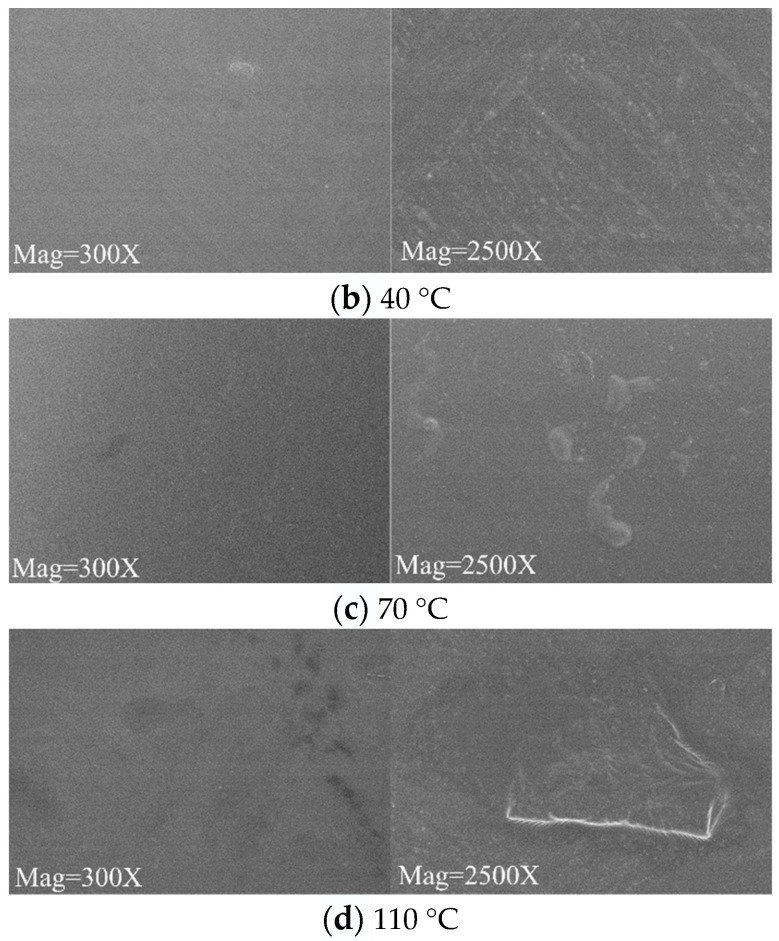
SEM of PET material.

In order to explore the changes in the surface morphologies of PD flexible UHF antenna sensor substrate materials, scanning electron microscopy was used to magnify the surface of PD flexible UHF antenna sensor substrate materials by 300 times and 2500 times before and after the experiment [23]. Since PDMS has low adhesion and cannot be stably fixed in the SEM sample stage, only the surface topographies of the PI and PET PD flexible UHF antenna sensor substrates were analyzed, and the results are shown in Figure 7 and Figure 8, respectively.

It can be seen from Figure 7 and Figure 8 that in the SF_6_/N_2_ mixed gas environment, when the magnification is 300 times, the surfaces of the PI and PET materials are smooth at three temperatures, and there is no obvious corrosion phenomenon. When the magnification is 2500 times, because the test points of the PI and PET materials are different at different temperatures, the surface morphologies of the PI and PET materials are different at different temperatures. The PI and PET materials have relatively few surface impurities in the mixed gas environment of 40 °C SF_6_/N_2_, and the PI and PET materials have comparatively many surface impurities in the mixed gas environment of 70 °C and 110 °C SF_6_/N_2_, among which, the surface impurities of the PI materials and PET materials show the shape of small islands in the mixed gas environment of 110 °C SF_6_/N_2_. Since the surfaces of the PI and PET materials show impure morphologies in three different temperature environments of 40 °C, 70 °C and 110 °C, and there is no obvious corrosion phenomenon, it can be concluded that neither PI nor PET reacts with SF_6_/N_2_ mixed gas.

#### 4.2.2. PD Flexible UHF Antenna Sensor Substrate Material Surface Element Detection

**Figure 9:**
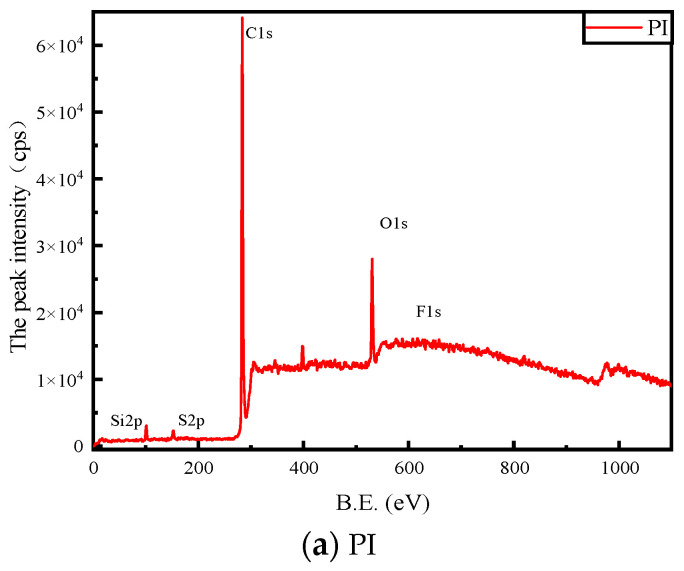

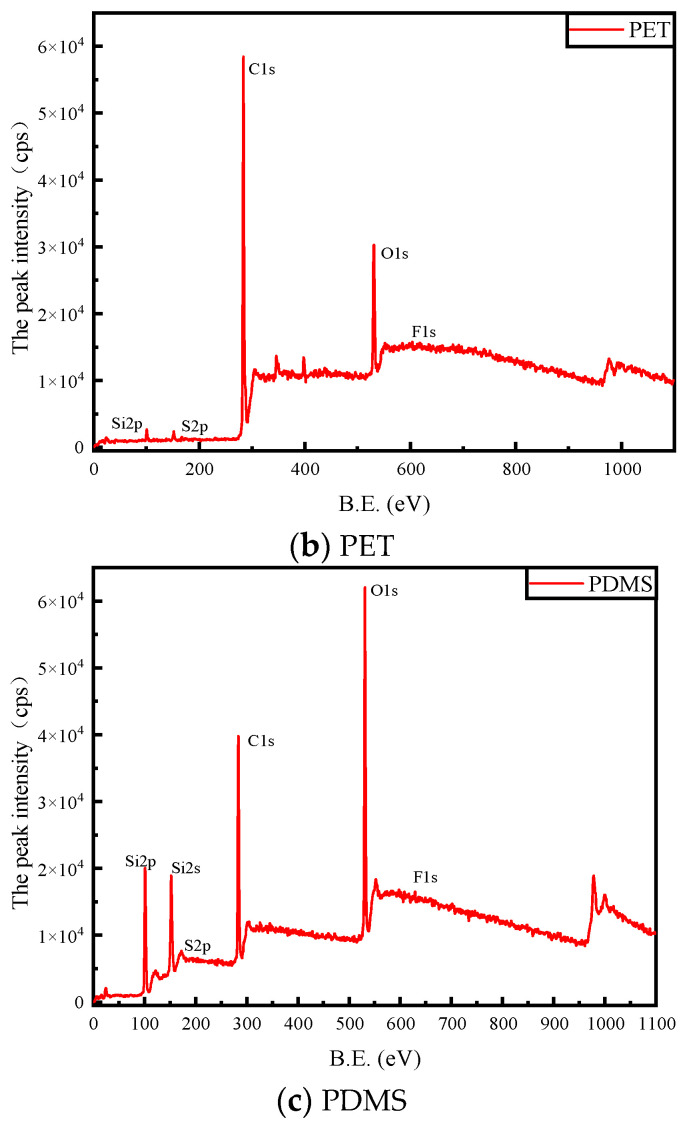
Photoelectron spectra of the full spectrum of elements before PI, PET and PDMS material experiments.

In order to analyze the possible changes in the substrate material surface of the PD flexible UHF antenna sensor, XPS analysis of the possible elements (C1s and F1s) on the sample surface was carried out. The detected absorption peaks were analyzed with Multipak software, and Shirley-type fitting subtraction was used to fit the peaks with the Gauss algorithm, where the charge calibration element was C1s (284.8 eV) [24,25]. The full spectra of elements on the substrates of three kinds of PD flexible UHF antenna sensors (PI, PET and PDMS) before the experiment are shown in Figure 9; from the figure, it can be seen that the substrates of three kinds of PD flexible UHF antenna sensors, PI, PET and PDMS, mainly contain C, O and S elements and contain trace amounts of F elements.

**Figure 10:**
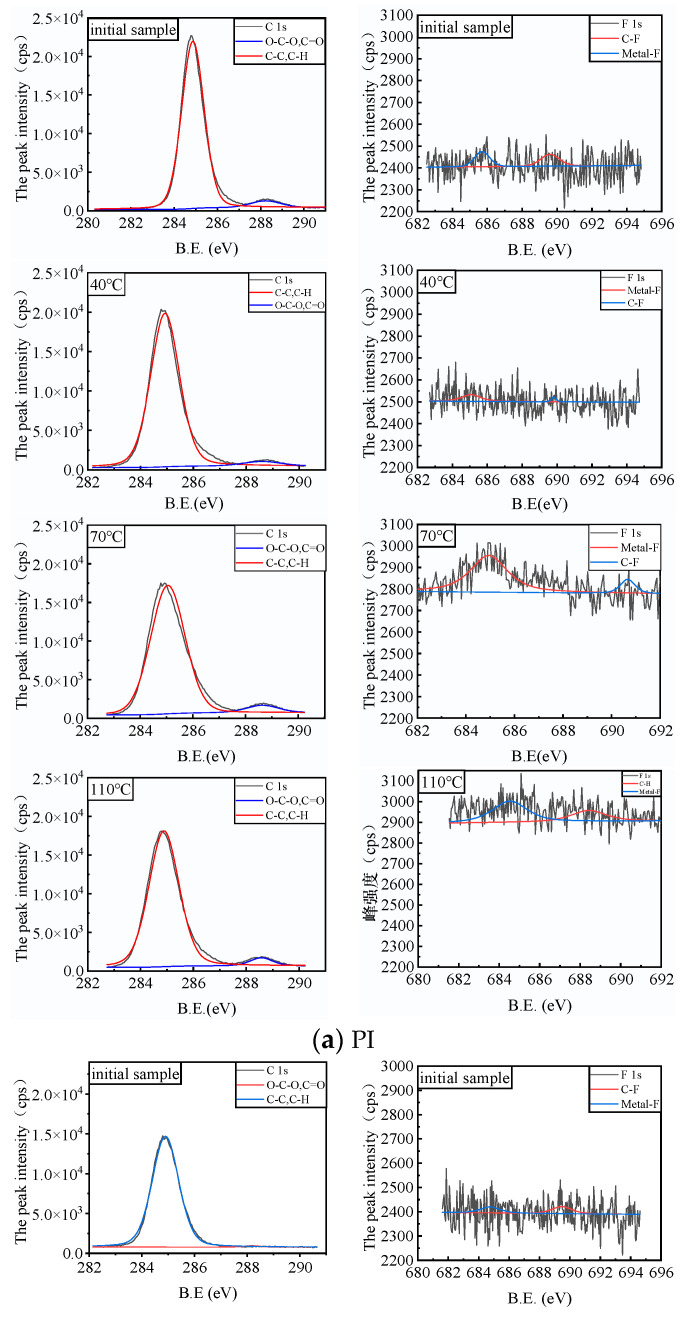

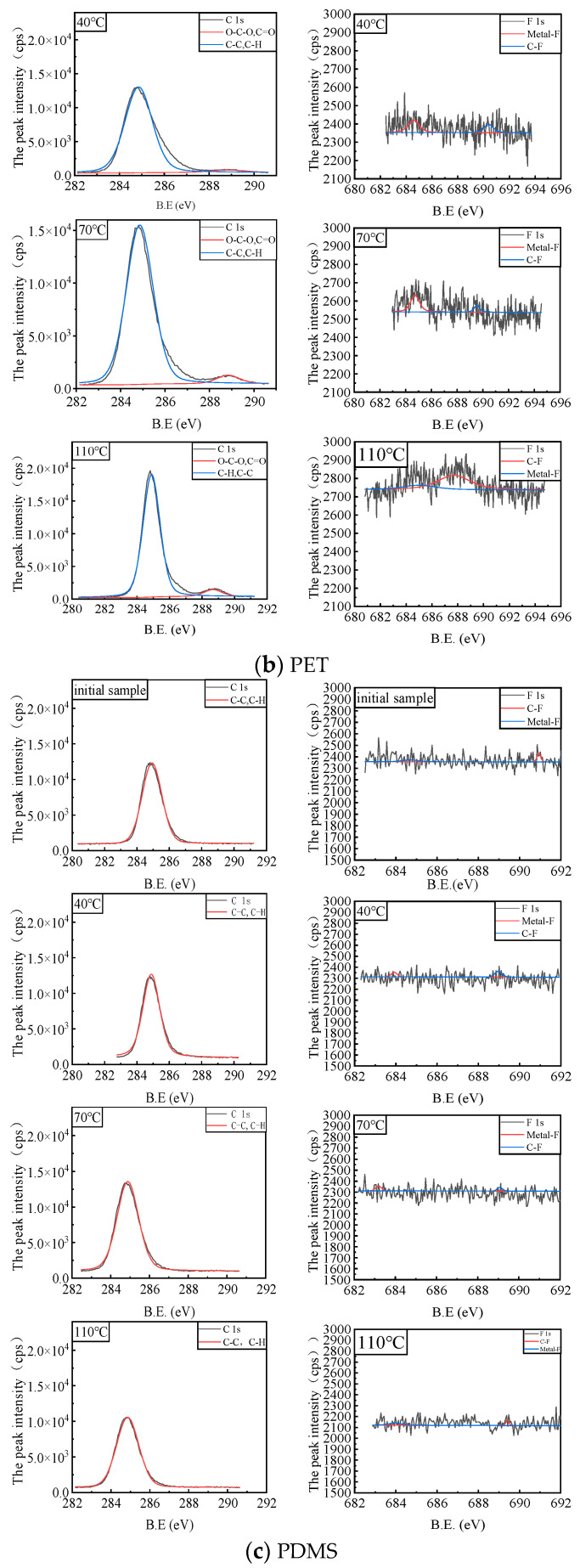
Fine spectra of surface elements of PI, PET and PDMS before and after the experiment.

Figure 10 shows the high-resolution photoelectron spectra of C1 and F1 elements before and after experiments on three kinds of PD flexible UHF antenna sensor substrates: PI, PET and PDMS. It can be seen from Figure 10a,b that the C1s of the PI and PET materials before and after the experiment detected characteristic peaks of C-C, C-H bonds and C=O, O-C-O bonds at 284.8 eV and 288.5 eV. It can be seen from Figure 10c that the C1s of the PDMS materials before and after the experiment only detected the characteristic peaks of C-C and C-H bonds at 284.8 eV. It can also be seen from Figure 10 that the content of C1s of PI, PET and PDMS did not change significantly with the increase in the experimental temperature in the SF_6_/N_2_ mixed gas environment at different temperatures of 40 °C, 70 °C and 110 °C. At the same time, by observing Figure 10b, it is not difficult to see that the absorption peaks of C=O and O-C-O bonds of PET materials in the 110 °C SF_6_/N_2_ mixed gas environment are higher than those of C=O and O-C-O bonds in the 40 °C and 70 °C SF_6_/N_2_ mixed environment. At the same time, the characteristic peaks (C=O and O-C-O bond) of C1s at the binding energy of 288.5 eV also increased with the increase in the experimental temperature, indicating that PET surface oxidation occurred under the action of the SF_6_/N_2_ gas mixture.

In addition, it can be seen from Figure 10a that when PI is in the 70 °C and 110 °C SF_6_/N_2_ mixed gas environments, F1s detects metal-F bonds and C-F bonds at the binding energy of 684.5 eV and 688.15 eV, respectively, indicating that the PI material reacts with the SF_6_/N_2_ mixed gas. Metal fluoride is formed, and the newly generated metal fluorine compounds are adsorbed on the surface of the PI material, resulting in the accumulation of element F on the surface of the PI material. At the same time, the detection of C-F bonds on the surface of the PI material indicates that the PI material reacts with components in the mixed gas, so that the F element replaces the H element in the PI material, and a new fluorine-containing compound is generated. At the same time, the newly generated fluorine-containing compound is adsorbed on the surface of the PI material, resulting in the accumulation of the F element on the surface of the PI material. At the same time, it can also be seen from Figure 10a that the peak intensity of F1s did not change significantly with the increase in the experimental temperature. As can be seen from Figure 10b, F1s also detected metal-F and C-F bonds at the binding energy of 684.5 eV and 688.15 eV. However, with the increase in the experimental temperature, the peak strength of the metal-F bond and C-F bond gradually increased, indicating that more fluoride accumulated on the surface of the PET material with the increase in temperature. As can be seen from Figure 10c, when PDMS was under three different-temperature SF_6_/N_2_ gas mixtures, F1s did not detect characteristic peaks, indicating that PDMS would not react with the SF_6_/N_2_ gas mixtures. Through the above analysis, it can be concluded that the PDMS material has good compatibility with SF_6_/N_2_ gas mixtures.

The results show that PDMS has good compatibility with SF_6_/N_2_ gas mixtures.

## 5. UHF Antenna Sensor Based on PDMS

This research group developed a flexible Hilbert antenna and a flexible planar bicone antenna based on PDMS. In reference [26], the flexible Hilbert antenna designed by our research group could reach 73.6% coverage at frequencies of 422–800 MHz, 1.23–1.58 GHz, 1.65–1.7 GHz and 1.79–3 GHz without bending, and bending did not affect the antenna bandwidth. When the curvature of the antenna was low, the cross polarization component of the antenna was less in the low frequency band, showing a better polarization effect, while the influence of the curvature was more obvious in the high frequency band, and some curvature would increase the cross polarization ratio. When the bending radius of the antenna was 350 mm, the peak gain of the antenna reached 1.34 dB. In the PD detection band, the radiation efficiency of the antenna with different bending radii was 30~50%. The flexible planar bipyramidal antenna designed by the research group in the literature [27] was divided into a finite lateral branch plane bipyramidal antenna and an infinite lateral branch plane bipyramidal antenna. The return loss characteristics of the two antennas were less than −10 dB in the 0.3–0.5 GHz, 0.64–1.25 GHz and 1.4–3.0 GHz bands. Meanwhile, the two planar double-cone antennas had good bandwidth and gain under different bending radii of 0 mm, 150 mm, 350 mm and 500 mm. The infinite lateral branch plane bipyramidal antenna could achieve a gain of 5.38 at 1.37 GHz. The finite lateral branch-plane bipyramidal antenna had stable radiation performance at 0.3–3 GHz, and the infinite lateral branch-plane bipyramidal antenna had stable radiation performance at 0.5 GHz–3 GHz. Both antennas could effectively detect UHF PD signals.

## 6. Conclusions

In order to investigate the compatibility of the substrate material of a GIS PD flexible UHF antenna sensor with SF_6_/N_2_ mixed gas, this paper built an experimental platform to determine the compatibility of SF_6_/N_2_ gas mixtures with substrates of PD flexible UHF antenna sensors to study the compatibility of SF_6_/N_2_ gas mixtures with the flexible antenna materials at different temperatures; through the analysis of the experimental results, the following conclusions are drawn:

(1) The composition of the SF_6_/N_2_ mixture was almost the same before and after the compatibility experiment, and the substrate materials of PI, PET and PDMS had no influence on the composition of the SF_6_/N_2_ mixture.

(2) PET slightly oxidized at 110 °C 30% SF_6_/70% N_2_, and the metal fluoride formation of PI was observed at 30% SF_6_/70% N_2_.

(3) The PDMS substrate is suitable for the development of flexible UHF antenna sensors.

## Data Availability

The data presented in the article is original and has not been inappropriately selected, manipulated, enhanced or fabricated by us.
